# Visualizing Autophagic Flux during Endothelial Injury with a Pathway-Inspired Tandem-Reaction Based Fluorogenic Probe

**DOI:** 10.7150/thno.33867

**Published:** 2019-08-01

**Authors:** Yu Lei, Wenming Ren, Cheng-Kun Wang, Rong-Rong Tao, Huai-Jiang Xiang, Li-Li Feng, Yin-Ping Gao, Quan Jiang, Xin Li, Youhong Hu, Feng Han

**Affiliations:** 1 College of Pharmaceutical Sciences, Zhejiang University, Hangzhou 310058, China; 2 State Key Laboratory of Drug Research, Shanghai Institute of Materia Medica, Chinese Academy of Sciences, Shanghai 201203, China; 3 School of Pharmacy, Nanjing Medical University, Nanjing 211166, China

**Keywords:** Fluorescent imaging, fluorescent probe, autophagy, endothelial injury

## Abstract

Autophagy is a dynamic and complicated catabolic process. Imaging autophagic flux can clearly advance knowledge of its pathophysiology significance. While the most common way autophagy is imaged relies on fluorescent protein-based probes, this method requires substantial genetic manipulation that severely restricts the application. Small fluorescent probes capable of tracking autophagic flux with good spatiotemporal resolution are highly demanable.

**Methods:** In this study, we developed a small-molecule fluorogenic probe (**AFG-1**) that facilitates real-time imaging of autophagic flux in both intact cells and live mice. **AFG-1** is inspired by the cascading nitrosative and acidic microenvironments evolving during autophagy. It operates over two sequential steps. In the first step, **AFG-1** responds to the up-regulated peroxynitrite at the initiation of autophagy by its diphenylamino group being oxidatively dearylated to yield a daughter probe. In the second step, the daughter probe responds to the acidic autolysosomes at the late stage of autophagy by being protonated.

**Results:** This pathway-dependent mechanism has been confirmed first by sequentially sensing ONOO^-^ and acid in aqueous solution, and then by imaging autophagic flux in live cells. Furthermore, **AFG-1** has been successfully applied to visualize autophagic flux in real-time in live mice following brain ischemic injury, justifying its robustness.

**Conclusion:** Due to the specificity, easy operation, and the dynamic information yielded, **AFG-1** should serve as a potential tool to explore the roles of autophagy under various pathological settings.

## Introduction

Autophagy is a homeostatic intracellular degradation process that serves to control protein quality 1-3. Dysregulation of autophagy can lead to cellular dysfunction and even cell death. Despite evidence indicating that autophagy is linked to a wide variety of prominent diseases including cancer 4, stroke 5, and neurodegeneration 6, very little is known about the cellular pathologic mechanisms 7. To assess the translational relevance in treating autophagy-related diseases, it is vital to have a reliable assay to detect autophagic flux under pathological contexts. Immunostaining assays have been widely used to detect autophagy, but they are limited to fixed tissues or homogenates and cannot be used in live cells 8. Electron microscopy is another widely used tool for autophagy detection, but it needs considerable specialized expertise and precludes functional studies 9. Optical imaging of autophagy is not only technically more accessible, but may facilitate functional studies. Optical imaging has therefore become the method of choice in autophagy study. Currently, optical imaging of autophagy is usually realized by the expression of fluorescent fusion proteins 10. However, this requires genetic manipulation and is technically highly demanding. Moreover, the incorporation of the non-native big fusions may affect the normal cellular biological process. On the contrary, bio-imaging employing small-molecule probes is not only technically more easily accessible, but poses little interference with normal biological process. Small-molecule probes are therefore more desirable as imagine tools. Actually, several small-molecule probes have been reported to facilitate autophagy imaging. For example, markers of acidic organelles have been used to visualize autophagy due to the acidic feature of autolysosomes 11-13. But these markers are only limited to detect the late stage of autophagy. Autophagy may also be visualized employing probes targeting reactive oxygen species (ROS), as ROS overproduction is involved in the autophagic process 14. Nevertheless, due to the extensive involvement of ROS in a wide range of pathophysiology processes other than autophagy, this method is inherently limited by its compromised specificity. Therefore, more reliable probes with improved specificity and sensitivity, and capable of tracing the dynamic autophagic flux in live specimens remain highly demanding.

Herein, we report a small-molecule fluorogenic probe for visualizing the autophagic flux both* in vitro* and *in vivo*. The probe is designed based on the characteristic of the autophagic pathway. In the early stage of autophagy, its fluorescence is switched on by the up-regulated peroxynitrite via oxidative dearylation, while in the late stage of autophagy, the daughter probe is protonated by the surrounding acidity to induce a further enhancement of the fluorescence intensity. This pathway-dependent design rationale has been validated by the reliability of probe **AFG-1** to record the autophagic flux during endothelial injury in both live cells and mice with high spatiotemporal resolution.

## Experimental section

### Probe synthesis and characterization

These procedures and details are included in the Supplemental Information.

### Cell culture

EA.hy926 cell line was used in this study. Cells were cultured in DMEM culture medium with 10% FBS in the 37°C incubator containing 5% CO_2_ and certain humidity. To induce autophagy or to inhibit autophagy, cells were exposed to HBSS, HBSS with 3-MA (1 mM), or HBSS with Bafilomycin A1 (100 nM). Cells without treatment served as the control.

### Confocal fluorescence staining and analysis

The RFP-LC3 plasmid was transfected into EA.hy926 endothelial cells for 48 h before the cells were cultured on glass cover slips overnight. Cells were then treated with HBSS for 3.5 h. After that, the media was changed to fresh HBSS containing **AFG-1** (50 nM) and a further incubation of 30 min was allowed. Cells were then fixed with 4% PFA. Nuclei were stained with 4', 6-diamidino-2-phenylindole (DAPI, Sigma). Intracellular probe fluorescence was visualized by confocal microscopy (Nikon A1R) and intensity was analyzed using Image J software (NIH, Bethesda, MD, USA).

For time-lapse confocal imaging of live cells, endothelial cells were transfected with RFP-LC3 plasmid and then cultured on glass-bottom 6-well plates overnight. After cells were incubated with **AFG-1** (50 nM) at 37 °C for 30 min, the dynamic change of **AFG-1** and LC3 fluorescence with or without HBSS treatment was captured every minute by time-lapse confocal microscope for 1 h.

### Animals and Adenovirus brain injection

Adult (2-3 months old) male C57BL/6 mice were used and were housed on a 12 h light/dark cycle at a constant temperature of 22 ± 1 °C with 40-60% humidity. All animal studies were approved by the Committees for Animal Experiments of Zhejiang University in China and conform to NIH guidelines (Guide for the Care and Use of Laboratory Animals. NIH publication no. 85-23, revised 1996). Adenovirus-RFP-LC3 was injected into the cortex of the mice. Photothrombosis ischemia model was performed 6 days post-injection.

### Photothrombosis-induced focal ischemia in mice

Animals were anesthetized with chloral hydrate at a dosage of 400 mg/kg, then mounted on the stereotaxic apparatus. Body temperature was maintained with a heating pad. The thrombotic occlusion of the cortex vessels was induced by the photochemical reaction. An incision was made over the skull. Rose bengal (10 mg/mL solution in saline, Sigma Chemical Co, St Louis, Mo) was injected intraperitoneally (ip, 0.1 mL/10g) 5 min before laser illumination. For illumination, a fiber-optic bundle of a cold light source (Nikon, QXA 10504 LCD, 21V 150W) was used. The periosteum over the skull was removed completely and the cold light source was placed close to the skull. The brain was illuminated for 20 min through the exposed intact skull. Then the skin was sutured, and animals were kept in a heated recovery box until awake.

### Open skull operation and probe incubation

At 24 h after the induction of photochemically induced thrombosis stroke, a craniotomy window was prepared in anesthetized mice. **AFG-1** (0.05 μM) was dissolved in DMSO and diluted in artificial cerebrospinal fluid (CSF), and was slowly injected (2 µL/s × 3s, delay 30 s, total volume 0.54 µL) into cortex at a depth of 100-300 μm below the cortical surface by standard patch-clamp pipette. Then the craniotomy window was covered by a removable cover glass lid (diameter 6.0 mm). The space between the exposed brain surface and the cover glass was filled with 1.5% (w/v) low melting point agarose in an artificial CSF. A metal frame of diameter 10.0 mm with the cover glass lid was glued to the skull to cover the craniotomy window. A bolus of 5 mg/kg Texas Red dextran (70 kDa, Molecular Probes, Invitrogen, Carlsbad, California) in 0.9% NaCl was injected into the tail vein to map the brain vessels before two-photon laser scanning microscope (TPLSM) imaging.

## Results and Discussion

### Probe Design and Synthesis

To develop a probe capable of monitoring the autophagic flux with high specificity, we first focused our attention on the chemical basis of the autophagic process. Our previous results show that nitrosative stress induces autophagy in endothelial cells 15, which is in accord with other reports that up-regulated ROS induces autophagy 16. Once autophagy is initiated, the newly formed autophagosomes become another major site for basal oxidative species generation 17. After engulfing cytoplasmic materials and damaged organelles, autophagosomes fuse with lysosomes to form autolysosomes which are endowed with both acidic pH levels and hydrolases for cargo degradation. Therefore, the cascading emergence of oxidative species and acidic interstitium may be considered as a potential biomarker for autophagy. Fluorescent probes that can sequentially respond to autophagy-related oxidative species and acidic pH levels may have desirable specificity for monitoring the autophagic process as a whole.

**Figure 1:**
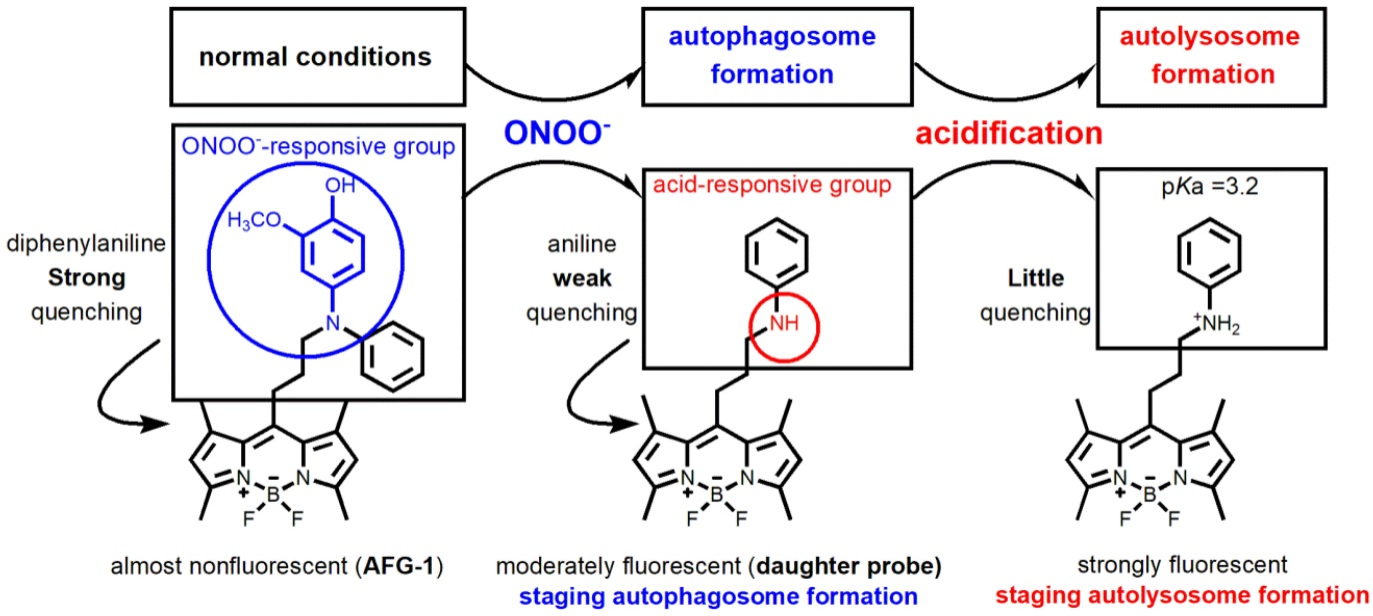
** Design of probe AFG-1 inspired by the characteristic of the autophagic pathway.** In the initial stage of autophagy, the up-regulated ONOO^-^ oxidizes the electron-rich aryl group (blue) into a quinone which is then cleaved following hydrolysis, yielding the daughter probe accompanied by a fluorescent switch-on response. As autophagy proceeds and autolysosomes form, the increased acidity in the microenvironment protonates the daughter probe, causing a further fluorescence enhancement.

The nitrosative stress marker, peroxynitrite (ONOO^-^), has been implicated in the initiation of autophagy in endothelial cells and may work as the first target for imaging autophagy 15,18. Among the various probes reported to image ROS 19-21, those that are designed based on the dearylation of electron-rich diphenylamines into mono-aryl-anilines attracted our interest due to their specifcity towards ONOO^-^ 22-25. Another merit of employing a diphenylamino group as the sensing trigger lies in its function as a good fluorescence quencher. It is reported that an unsubstituted diphenylamino group usually demonstrates a stronger fluorescence quenching effect than an unsubstituted phenylamino group 26. While obviously, diphenylamines should show weaker basicity than their dearylated aniline products. Based on these facts, we reason that ONOO^-^ -sensitive diphenylamines should work as an ideal sensing trigger for the autophagic flux. Tailored diphenylamines should respond to ONOO^-^ at the early stage of autophagy via the oxidative dearylation reaction to yield the aniline that is more acidotropic. The resulting aniline should sequentially respond to the acidified microenvironment in the late stage of autophagy by being protonated (Figure 1). Based on these considerations, we designed probe **AFG-1** by incorporating a well-tailored diphenylamino group into the BODIPY fluorophore which features two-photo excitation properties 27. We reason that **AFG-1** should be of no fluorescence due to the quenching effect of the diphenylamino group. However, at the early stage of autophagy, a burst of ONOO^-^ should switch on its fluorescence by oxidative dearylation to transform **AFG-1** into the daughter probe. As the autophagic process proceeds and autolysosomes are formed at the late stage of autophagy, the daughter probe is protonated and a further fluorescence enhancement should be observed (Figure 1). Facilitated by this two-step activation mechanism, **AFG-1** may realize the specific observation of the autophagic flux in real time.

Probe **AFG-1** was synthesized *via* reductive amination of 1, 3, 5, 7-tetramethyl-8- (3-oxopropyl) BODIPY with 2-methoxy-4-(phenylamino)phenol. Detailed procedures were described in the supporting information.

### Fluorescence response of AFG-1 to the cascading ONOO^-^ and acid in aqueous solution

**Figure 2:**
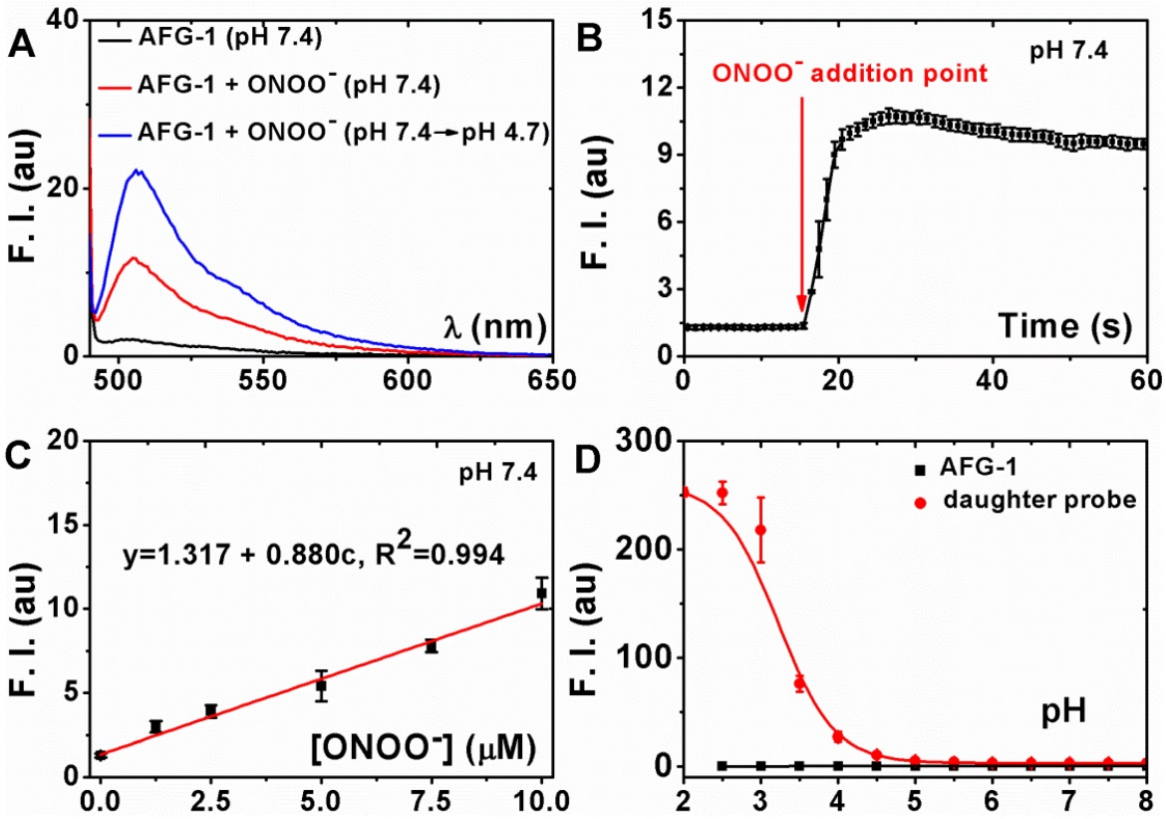
** Characterization of the fluorogenic response of AFG-1 to the subsequent treatment of ONOO^-^ and acid. A) AFG-1** (5 μM) was first treated with ONOO^-^ (10 μM) for 30 min and then the solution was acidified to pH 4.7 to record the fluorescent spectra. **B)** The emission intensity of **AFG-1** (5 μM) at 505 nm was recorded as time lapsed. During the recording, ONOO^-^ (10 μM) was quickly added to further record the response. **C) AFG-1** (5 μM) was first treated with various concentrations of ONOO^-^ and then the spectra were recorded. Data shown were the plot of the intensity at 505 nm against ONOO^-^ concentration. **D)** Both **AFG-1** and the daughter probe were dissolved in PBS (with 10% ethonal) of various pH values to record the fluorescence spectra. Data shown were the plot of their emission intensity at 505 nm against surrounding pH values. All data were obtained on a Cary Elipse spectrofluorimeter with slit widths to be 2.5 and 5 nm (A, B, C) or 2.5 and 2.5 nm (D) for excitement and emission respectively.

To evaluate the ability of probe **AFG-1** to sense sequentially appearing ONOO^-^ and acid, its fluorescent spectra under indicated conditions were recorded in phosphate buffer solution (PBS, 100 mM, with 10% ethonal). **AFG-1** was almost non-fluorescent at pH 7.4 (Figure 2A). Treating **AFG-1** with two equivalents of ONOO^-^ induced a 7-fold fluorescent intensity enhancement (Figure 2A), suggesting the transformation of **AFG-1** to the daughter probe in response to ONOO^-^ stimulation. The production of the daughter probe was also confirmed by tracking the reaction by LCMS (Figure S1-S3). When this solution was acidified to pH 4.7, a further fluorescent intensity enhancement of 7-fold could be observed (Figure 2A), indicating the protonation of the daughter probe in response to acidification. These observations indicate that the fluorescence of **AFG-1** can indeed be activated by a cascading stimulation of ONOO^-^ oxidation and acid protonation. We also confirmed that the fluorogenic response of **AFG-1** was selective towards ONOO^-^ among a panel of reactive species commonly found in biological system (Figure S4).

The fluorescence quenching mechanism was also studied by a Gaussian calculation on the frontier molecular orbital (FMO) of the excited fluorophore, the diphenylaniline, the aniline and the protonated aniline moiety. Data were shown in Figure S5, which showed an acceptor-excited intramolecular photoinduced electron transfer (a-PeT) mechanism for **AFG-1** 28.

### Sensitivity of the fluorogenic response of AFG-1 to ONOO^-^ in aqueous solution

Having confirmed the tandem fluorescence-enhancement response of **AFG-1** to ONOO^-^ and acid, we then set out to test its sensitivity to ONOO^-^. For this purpose, the kinetic and stoichiometric characterization of the detection reaction was carried out in PBS (100 mM, with 10% ethonal). It turned out that the fluorescent intensity of **AFG-1** (5 µM) increased fast after ONOO^-^ (10 µM) treatment and plateaued in seconds (Figure 2B), suggesting that the detection reaction between **AFG-1** and ONOO^-^ favors a fast kinetics. Given this fast response of **AFG-1** to ONOO^-^, we suspect that the probe should be capable to track the burst of biological ONOO^-^ despite its elusive nature.

Next, **AFG-1** (5 µM) was treated with various doses of ONOO^-^ to check the ONOO^-^ -dose dependent responsive profile. It turned out that the fluorescent intensity of **AFG-1** increased in an ONOO^-^ -dose dependent way. The correlation happens to be linear with ONOO^-^ concentrations ranging from 0 to 10 μM (Figure 2C). Noteworthy, when **AFG-1** (5 µM) was treated with a concentration of ONOO^-^ higher than 20 μM, it resulted in a less potent fluorescent intensity enhancement (Figure S6, S7), suggesting that a large excess of ONOO^-^ may destroy the daughter probe presumably by the nitration / nitrosation of the aniline nitrogen atom 29. We hypothesize this as a desirable feature of **AFG-1** to guarantee its specificity to autophagy - if nitrosative stress proceeds without initiating autophagy, the overproduced ONOO^-^ would quench the fluorescence of the probe to avoid giving biased signals.

### Sensitivity of the daughter probe towards acidification in aqueous solution

To test the sensitivity of the daughter probe towards acid surroundings, an acid titration experiment was carried out to determine its p*K*a. When the emission intensity of the daughter probe (at 505 nm) in PBS (100 mM, with 10% ethonal) of various pH values was plotted against the solution pH values, the p*K*a of the daughter probe was measured to be 3.2 (Figure 2D). We think this p*K*a should make the daughter probe sensitive enough to image the maturation from autophagosomes (neutral pH) to autolysosomes (with an acidic pH). It should be noted that probe **AFG-1** alone is inert toward acidification (Figure 2D), further supporting the two-step activation mechanism of **AFG-1** by the cascading autophagic process.

### Monitoring autophagy in live endothelial cells

**Figure 3:**
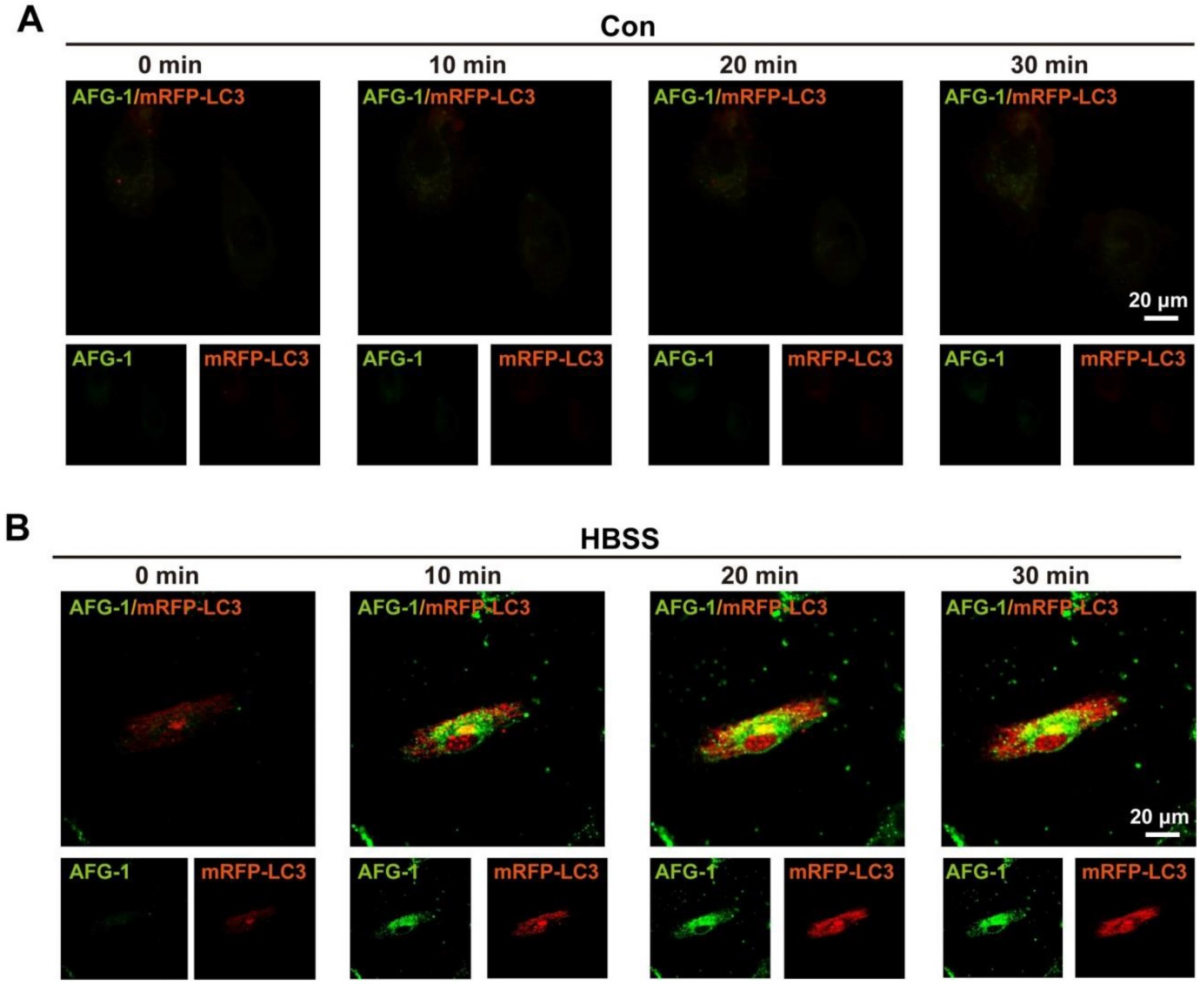
** Imaging autophagic flux in live endothelial cells.** Data shown were representative confocal images of probe **AFG-1** (green, λ_ex_ 488 nm, λ_em_ 505-550 nm) and mRFP-LC3 puncta (red, λ_ex_ 543 nm,λ_em_ 560-615 nm) in 1 min, 10 min, 20 min and 30 min time frame in control (**A**) and HBSS-treated endothelial cells (**B**). Scale bar: 20 μm.

Having confirmed the tandem response of **AFG-1** to the sequential treatment of ONOO^-^ and acid in aqueous solution, we moved on to test its feasibility to image autophagy in live cells. For this purpose, endothelial cells were selected because the endothelium has been reported to be one of the major site of excessive autophagy 30. **AFG-1** was first tested to cause no effect on cell viability (Figure S8). Cells were then transfected with mRFP-LC3 as a positive control since fluorescent protein-tagged LC3 is a general marker for autophagosomes 31. After subjecting the cells to HBSS-induced nutrient starvation to trigger on autophagy 32, the intracellular dynamic fluorescence change of **AFG-1** in response to HBSS-treatment was recorded. As shown in Figure 3B, the intracellular **AFG-1** fluorescence increased gradually following prolonged HBSS treatment and co-localized well with that of mRFP-LC3 (Pearson correlation coefficient, 0.433). While in contrast, no significant fluorescence intensity change was observed in control cells incubated under normal conditions (Figure 3A). This result highlights **AFG-1** as a reliable probe with desirable sensitivity and specificity for tracing the autophagic process. Noteworthy, **AFG-1** could still work well to track the autophagic process even after a prolonged HBSS treatment time of 12 h (Figure S10), highlighting the robustness of probe **AFG-1** for long-term tracking of autophagy process.

### Characterization of the pathway-dependent response of AFG-1 to the autophagic process in live cells

Having confirmed the feasibility of probe **AFG-1** to image the autophagic process in live endothelial cells, we then moved on forward to characterize its autophagy pathway-dependent response. Cells were subjected to genetic or pharmacological manipulation to intervene in the autophagy pathway, and the effect of these interventions on intracellular **AFG-1** fluorescence was recorded.

**Figure 4:**
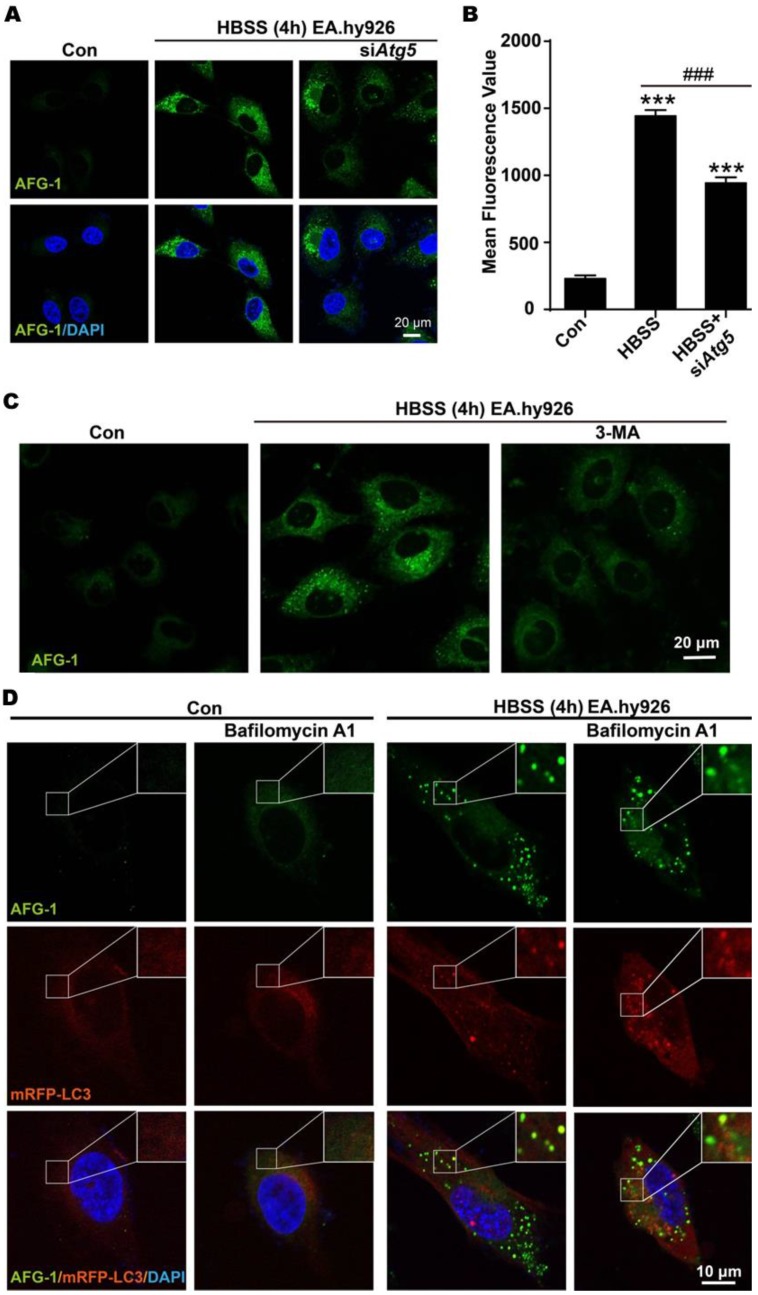
**Imaging early and late stages of autophagy with AFG-1. A)** Representative confocal images of **AFG-1** and DAPI in endothelial cells under normal or starvation conditions. Inhibiting *Atg5* with siRNA to block early stage autophagy resulted in weaker **AFG-1** fluorescence compared with the non-inhibiting group. **AFG-1** (50 nM) was incubated for 30 min. **B)** Quantitative analysis of **AFG-1** fluorescence in a, N=3 (4 - 6 fields were taken), ^***^P<0.001 with one-way ANOVA compared with control group, ^###^*P*<0.001 with one-way ANOVA compared with HBSS-4 h group. **C)** Representative confocal images of **AFG-1** and DAPI in endothelial cells under normal or starvation conditions. 3-MA (1 mM) significantly decreased **AFG-1** fluorescence intensity. **AFG-1** probe (50 nM) was incubated for 30 min, scale bar: 20 μm. **D)** Representative images of **AFG-1** and mRFP-LC3 puncta in EA.hy926 cells under normal or starvation conditions. Bafilomycin A1 (100 nM) treatment increased mRFP-LC3 fluorescence while decreased **AFG-1** fluorescence as a result of autolysosome-inhibition. Scale bar: 10 μm. **AFG-1** fluorescence was recorded with λ_ex_ 488 nm, λ_em_ 505-550 nm (green). DAPI fluorescence was recorded with λ_ex_ 405 nm, λ_em_ 420-480 nm (blue). mRFP fluorescence was recorded with λ_ex_ 543 nm, λ_em_ 560-615 nm (red).

First, autophagy inhibition at early stage was conducted to examine its effect on the switch-on response of probe **AFG-1**. For this purpose, *Atg5* was knocked down by siRNA to intervene autophagosome formation, as *Atg5* is essential for autophagosome elongation 33. It was observed that siRNA knockdown of *Atg5* markedly attenuated starvation-induced increase of **AFG-1** fluorescence (Figure 4A). Quantitative analysis of intracellular **AFG-1** fluorescence showed a 6-fold intensity enhancement after starvation-stimulation compared with the non-starvation control group. However, this starvation-induced **AFG-1** fluorescence attenuated significantly in *Atg5*-silenced cells (*P*<0.001, Figure 4B). This result demonstrates that inhibiting autophagosome formation would inhibit the fluorogenic response of probe **AFG-1**, suggesting the specificity of **AFG-1** towards autophagy. Similar observations were obtained when cells were treated with 3-methyladenine (3-MA, 1 mM) to block autophagosome formation *via* inhibiting class III PI3K (Figure 4C) 10. These results corroborated the specificity and sensitivity of **AFG-1** for autophagosomes.

Then, late-stage autophagy was inhibited by treating cells with Bafilomycin A1 to block fusion between autophagosomes and lysosomes 10. As shown in Figure 4D, HBSS treatment induced the fluorescence switch on of **AFG-1**, which colocalized well with mRFP-LC3 (Pearson correlation coefficient, 0.74). Inhibiting autolysosome formation with bafilomycin A1 (100 nM) led to the elevation of mRFP-LC3 fluorescence level. While intracellular **AFG-1** fluorescence was found less intensified compared with the non-Bafilomycin A1 treatment group, manifesting the blockage of the protonation process of the probe, which is in agreement with Bafilomycin A1-induced fusion blockage of autophagosomes with lysosomes. Moreover, the colocalization of **AFG-1** and mRFP-LC3 was decreased in the Bafilomycin A1 treatment group as shown by the decreased Pearson correlation coefficient of 0.51. All these observations agree well with the microenvironment features of autophagy, and further support the **AFG-1** probe design rationale.

### *In vivo* real-time imaging of autophagy in living mice after brain ischemia

**Figure 5:**
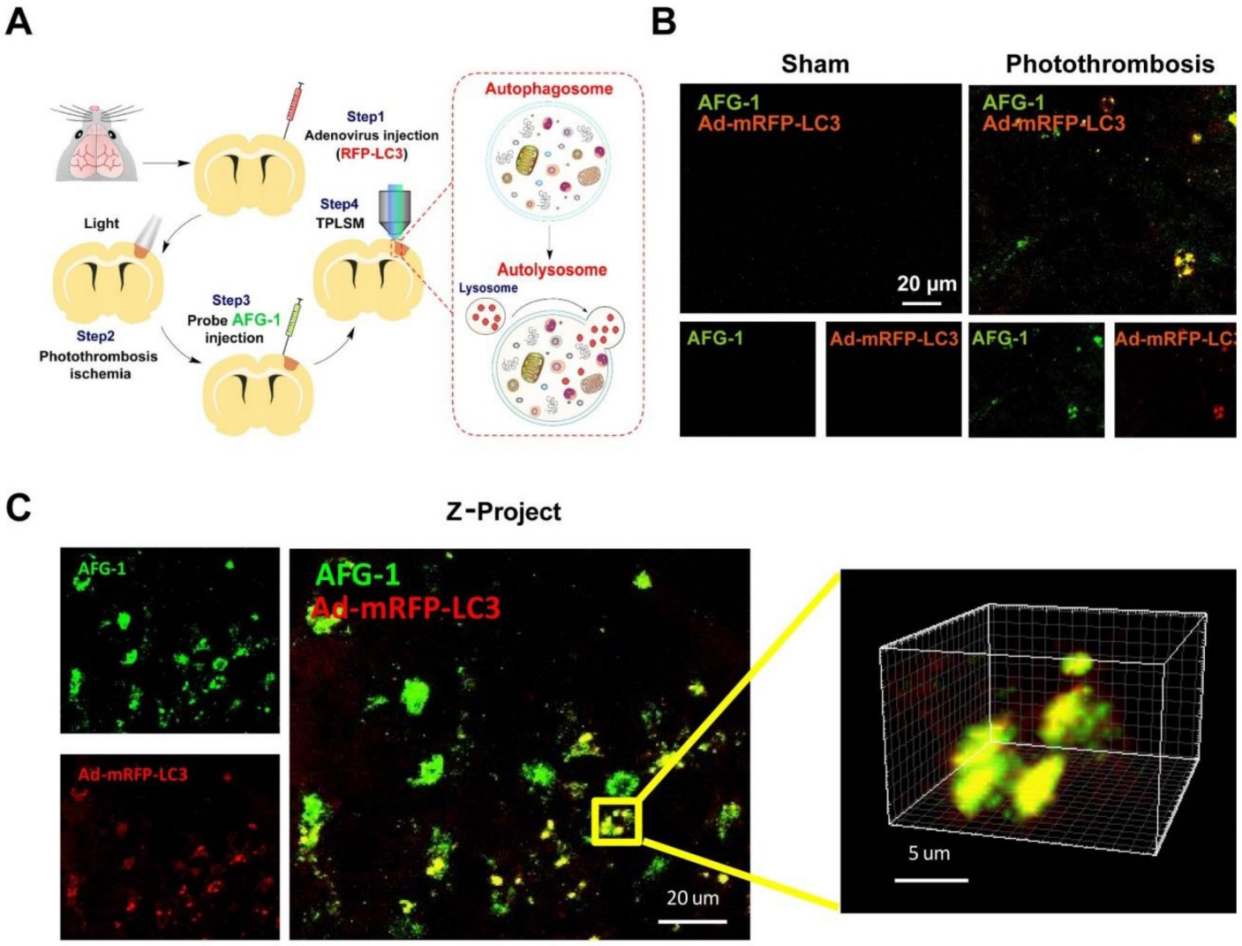
***In vivo* two-photon fluorescence imaging of autophagy using Probe AFG-1 and adenovirus-mRFP-LC3 following brain ischemia in live mice. A)** Schematic illustration of experimental procedures. **B)** Representative images of probe AFG-1 and adenovirus-mRFP-LC3 staining (red) in mice without or with photothrombosis. AFG-1 fluorescence elevated significantly after photothrombosis ischemia for 24 h, and colocalized well with mRFP-LC3. **C)** *Z*-project data for the colocalization of AFG-1 and the adenovirus-mRFP-LC3 in the ischemia brain, 150-200 µm below the cortical surface was selected for imaging. Scale bar: 20 µm. Emission was collected at 495-540 nm for AFG-1 fluorescence and 575-645 nm for mRFP upon two-photon excitation at 800 nm.

Having validated the capability of **AFG-1** to track the autophagic flux *in vitro*, we next set out to assess its performance in live mice. First, adenovirus carrying mRFP-LC3 was generated to work as a positive control to visualize autophagic vacuoles in neurovascular components in the brain. One week after adenovirus vector (5 μl, titer 5.0 × 10^6^ vector genomes/ml) injection into the brain cortex, the mice were investigated by two-photon microscopy (FV1000MPE2, Olympus) with the thin skull method as described previously 23, 34. Ischemia was induced by photothrombosis-induced vascular occlusion. Combined with *in vivo* two-photon laser scanning microscopy (TPLSM), **AFG-1** enabled visualization of the dynamic changes of the autophagic flux in penumbra region of live mice. The elevation of local autophagy vacuoles in the ischemic brain was indicated by strong **AFG-1** fluorescence, which was recorded over a period of 30 s (Figure 5, Movie S1). Consistent with our live cell imaging results, **AFG-1** fluorescence colocalized with the adenovirus-mRFP-LC3 staining (Red, Pearson correlation coefficient, 0.80) in neurovascular components of the brain following ischemia in living mice. While in contrast, no significant **AFG-1** fluorescence was observed in the control group without neurovascular insult (Movie S2). These results agreed well with our previous observation on vascular insult-induced ONOO^-^ overproduction 23, and provided the direct observation on ONOO^-^ -initiated autophagy activation upon ischemia injury.

## Conclusions

Inspired by the cascading microenvironment features of autophagy, we have developed a small-molecule fluorescent probe **AFG-1** for the specific visualization of the autophagic flux both *in vitro* and *in vivo*. The fluorescence of **AFG-1** can be activated first by ONOO^-^ stimulation and then by protonation under acidic context, which are two sequentially emerging microenvironmental features characterizing autophagy. This pathway-dependent responsive profile has been validated by imaging the autophagic process in live cells, which showed that intervening in the autophagic process with genetic or pharmacological manipulation accordingly affected the fluorescence response of **AFG-1**. Application of probe **AFG-1** to live mice imaging has provided the direct evidence on autophagy activation upon brain ischemia injury. Due to its sensitivity and the easy operation, probe **AFG-1** should serve as a powerful imaging tool to explore autophagy biology under a variety of pathological contexts *in vitro* and *in vivo*. Furthermore, this pathway-dependent probe design strategy as demonstrated in this work should be instructive for future probe design.

## Supplementary Material

Supplementary figures and tables.

Movie S1.

Movie S2.
