# Supplementary material for: Visualizing Autophagic Flux during Endothelial Injury with a Pathway-Inspired Tandem-Reaction Based Fluorogenic Probe
Source: Theranostics. 2019 Aug 1;9(19):5672–80. doi: 10.7150/thno.33867 (PMC6735386; doi:10.7150/thno.33867)
Supplement: Supplementary file 1 — Supplementary figures and tables. [file thnov09p5672s1.pdf]

SUPPORTING INFORMATION

Visualizing Autophagic Flux During Endothelial Injury with a Pathway-Inspired Tandem-Reaction Based Fluorogenic Probe

Yu Lei,[1,#] Wenming Ren,[2,#] Cheng-Kun Wang,[1] Rong-Rong Tao,[1] Huai-Jiang Xiang,[1] Li-Li Feng,[3] Yin-Ping Gao,[1] Quan Jiang,[1] Xin Li,[1,*] Youhong Hu,[2,*] Feng Han[1,3,*]

1. College of Pharmaceutical Sciences, Zhejiang University, Hangzhou 310058, China

2. State Key Laboratory of Drug Research, Shanghai Institute of Materia Medica, Chinese Academy of Sciences, Shanghai 201203, China

3. School of Pharmacy, Nanjing Medical University, Nanjing 211166, China

# These authors contributed equally

[*] Correspondence: lixin81@zju.edu.cn, yhhu@simm.ac.cn, fenghan169@njmu.edu.cn

Table of Contents

General experimental for chemistry 3

Probe synthesis and characterization 3

Preparation of $ONOO^-$ 5

General experimental for photophysical property characterization 5

Quantum Yields Determination 6

Table S1. 6

Supplementary Figures 7

- Figure S1 7
- Figure S2 8
- Figure S3 8
- Figure S4 9
- Figure S5 9
- Figure S6 10
- Figure S7 10
- Figure S8 11
- Figure S9 11

Supplementary Movies 12

- Movie S1 12
- Movie S2 12

^{1}H NMR and ^{13}C NMR spectra of AFG-1 13

^{1}H NMR and ^{13}C NMR spectra of the daughter probe. 14

General experimental for chemistry

Unless otherwise noted, all reagents were purchased from commercial suppliers and used without further purification. 1, 3, 5, 7-tetramethyl-8-(3-oxopropyl) BODIPY was prepared as we previously reported [1].

Anhydrous THF was distilled from Na prior to use. Reactions were monitored by thin layer chromatography using TLC Silica gel 60 F254 supplied by Qingdao Puke Seperation Meterial Corporation, Qingdao, P. R. China. Silica gel for column chromatography was 200-300 mesh and was supplied by Qingdao Marine Chemical Factory, Qingdao, P. R. China. Characterization of intermediates and final compounds was done using NMR spectroscopy and mass spectrometry. NMR spectra were recorded on Brucker AVANCE III 400 NMR spectrometer or Brucker AVANCE III 500 NMR spectrometer or Bruker Ascend 600 NMR spectrometer with d-CHCl3 as solvent and tetramethylsilane (TMS) as the internal standard. The following abbreviations were used to designate multiplicities: s=singlet, d=doublet, t=triplet, q=quartet, m=multiplet. All spectra were recorded at 25oC and chemical shifts were given in ppm and coupling constants (J) in Hz. High-resolution mass data were obtained on an Agilent G6520 Q-TOF LC/MS.

Probe synthesis and characterization

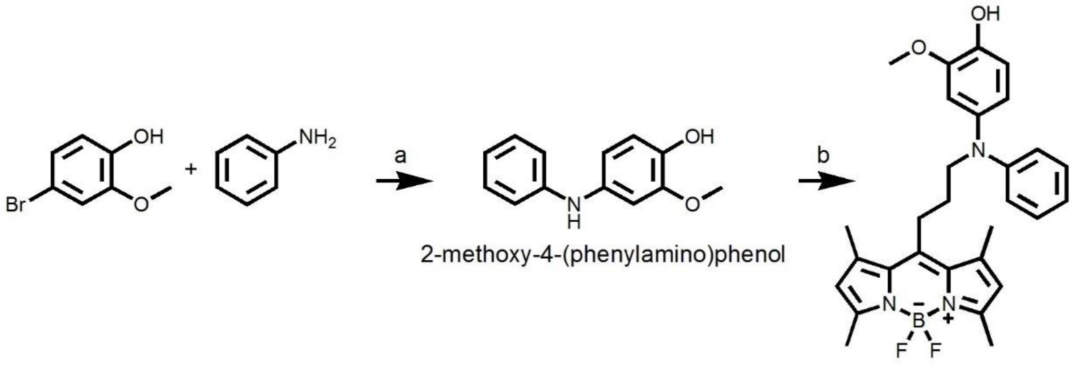

AFG-1

Reagents and conditions: (a) $Pd_2(dba)_3$, CyJohnPhos, $NaN(TMS)_2$, THF, 65°C, 42%; (b) 1, 3, 5, 7-tetramethyl-8-(3-oxopropyl) BODIPY, $NaBH_3CN$, AcOH (cat.), CH_2Cl_2/MeOH, ambient temperature, 15%.

---

1. Ren W, Xu M, Liang SH, Xiang H, Tang L, Zhang M, Ding D, Li X, Zhang H, Hu Y. Biosens Bioelectron. 2016; 75: 136-141.

Synthesis of intermediate "2-methoxy-4-(phenylamino)phenol"

This key intermediate was prepared according to literature procedures [2] To detail, 4-bromo-2-methoxyphenol (1.0 g, 4.9 mmol) was dissolved in anhydrous THF (50 mL), to which was added sequentially aniline (0.55 g, 5.9 mmol), $Pd_2(dba)_3$ (0.23 g, 0.25 mmol) and CyJohnPhos (0.21 g, 0.59 mmol) under an argon atmosphere. After the slow addition of $NaN(TMS)_2$ (7.4 mL of 2.0 M in THF, 15 mmol), the resulting mixture was warmed to 65°C and stirred for 6 h. The reaction mixture was cooled to ambient temperature and then poured into water (0.10 L), followed by neutralization with a saturated $KHSO_4$ solution. The mixture was extracted with ethyl acetate (2 × 50 mL). The combined organic layers were washed with brine (50 mL), dried over anhydrous Na_2SO_4, filtered, and concentrated to give the crude product, which was further purified by flash chromatography on a silica gel column eluted with a gradient of petroleumether/ethyl acetate (15:1-10:1) to afford compound 1 as a colorless oil (0.45 g, 42%).

^{1}H NMR (400 MHz, $CDCl_3$): δ 7.21 (t, J = 7.8 Hz, 2H), 6.90 (d, J = 7.8 Hz, 2H), 6.85 (d, J = 8.4 Hz, 1H), 6.83 (t, J = 7.8 Hz, 1H), 6.71 (d, J = 2.1 Hz, 1H), 6.64 (dd, J = 8.4, 2.1 Hz, 1H), 5.47 (s, 1H), 5.40 (s, 1H), 3.83 (s, 3H).

^{13}C NMR (150 MHz, $CDCl_3$): δ 146.94, 145.30, 141.41, 135.35, 129.36, 119.59, 115.68, 114.76, 114.06, 105.18, 77.26, 77.05, 76.84, 55.97.

HRMS (*m/z*): $[M+H]^+$ calcd for $C_{13}H_{14}NO_2^+$: 216.1019, Found: 216.1018.

Synthesis of probe AFG-1

Intermediate 2-methoxy-4-(phenylamino)phenol (0.12 g, 0.56 mmol) and 1, 3, 5, 7-tetramethyl-8-(3-oxopropyl) BODIPY (0.20 g, 0.67 mmol) were dissolved in a mixture of 1, 2-dichloroethane and methanol (5:1, 12 ml), followed by the treatment of $NaBH_3CN$ (42 mg, 0.67 mmol) and one drop of acetic acid. The resulting mixture was stirred at ambient temperature for 6 h. After evaporation of the volatile fraction, the residue was purified by flash chromatography on a silica gel column eluted with a gradient of petroleumether/ethyl acetate/CH_2Cl_2 (from 20:1:1 to 10:1:1) to afford **AFG-1** as a red solid (43 mg, 15%).

^{1}H NMR (400 MHz, $CDCl_3$): δ 7.20-7.16 (m, 2H), 6.90 (d, J = 8.4 Hz, 1H), 6.78 (t, J = 7.9 Hz,

2. Harris MC, Huang X, Buchwald SL. Org Lett. 2002; 4: 2885-2888.

1H), 6.74 (d, J = 7.9 Hz, 2H), 6.67 (dd, J = 8.4, 2.3 Hz, 1H), 6.64 (d, J = 2.3 Hz, 1H), 6.03 (s, 2H), 5.51 (s, 1H), 3.84 – 3.76 (m, 5H), 3.04 – 2.97 (m, 2H), 2.50 (s, 6H), 2.33 (s, 6H), 2.04 – 1.94 (m, 2H).

^{13}C NMR (125 MHz, $CDCl_3$): δ 154.74, 149.45, 147.89, 146.14, 143.62, 140.91, 132.06, 129.82, 122.43, 119.49, 119.31, 116.60, 115.70, 110.04, 56.70, 53.32, 30.50, 26.87, 16.99, 15.16.

HRMS (*m/z*): $[M+H]^+$ calcd for $C_{29}H_{33}BF_2N_3O_2^+$: 504.2641. Found: 504.2628.

Preparation of $ONOO^-$

To a vigorously stirred solution of $NaNO_2$ (0.6 M, 10 mL) and H_2O_2 (0.7 M, 10 mL) in deionized H_2O at 0^oC was added HCl (0.6 M, 10 mL), immediately followed by the rapid addition of NaOH (1.5 M, 20 mL). Excess hydrogen peroxide was removed by passing the solution through a short column of MnO_2. The concentration of $ONOO^-$ was determined by UV analysis with the extinction coefficient at 302 nm (ε= 1670 M^{-1} cm^{-1}). Aliquots of the solution were stored at -20 ^oC.

General experimental for photophysical property characterization

All the photophysical characterization experiments were carried out at an ambient temperature. Absorption spectra were acquired using a Hitachi U-3010 spectrophotometer. Fluorescence measurements were performed on a Cary Elipse spectrofluorimeter with slit widths to be 2.5 and 5 nm for excitement and emission respectively except otherwise indicated, and the sensitivity of the instrument was kept at medium.

Deionized water was used to prepare all aqueous solutions. Phosphate buffer saline (PBS, 100 mM, pH 7.4) was purged with N_2 for 5 min before use. The probe was dissolved in EtOH to make a 50 μM stock solution.

To test the fluorescent responses of the probe towards $ONOO^-$, aliquots of the probe stock solution were diluted with PBS and treated with $ONOO^-$ to make sure both the probe and $ONOO^-$ were kept at desired final concentrations. After quick and vigorous shaking, the mixture was allowed standing in the dark for desired time and the fluorescence spectra were then recorded under excitation at 488 nm except otherwise indicated. The emission spectra were scanned from 490 to 650 nm. All fluorometric experiments were performed in triplicate, and data shown were

the average.

For the pH titration experiments after treating **AFG-1** with $ONOO^-$, aliquots of the probe stock solution were diluted with PBS (100 mM, pH 7.4) and treated with $ONOO^-$ for 30 min (probe and $ONOO^-$ final concentrations were 5 μM and 10μM, respectively). Slow addition of HCl (1.0 M) then brought the solution to various pH values which were monitored by pH meter. Concentration change caused by the addition of HCl was neglected.

Quantum Yields Determination

UV absorption for **AFG-1** and the $ONOO^-$-sensing product probe were analyzed in PBS (100 mM, 10% EtOH, pH 7.4 and 4.0). Their quantum yields were determined using fluorescein ($\Phi_{standard}$ = 0.95 in 0.1 M NaOH) as a standard according to a published method with the following equation [3].

$$\phi_{sample} = \phi_{standard} \cdot \frac{Abs_{standard} \cdot \sum F_{sample}}{Abs_{sample} \cdot \sum F_{standard}} \cdot \frac{n_{sample}{}^2}{n_{standard}{}^2}$$

where Φ is the quantum yield, ΣF is the integrated fluorescence intensity, Abs is absorbance at λex 405 nm, and n represents the refractive index of the solvent.

Table S1. Photophysical properties of **AFG-1** and the $ONOO^-$ **-**sensing product (daughter probe) in comparison with a control compound. Data were obtained in PBS (100 mM, 10% EtOH, pH 7.4 or 4.0) at ambient temperature.

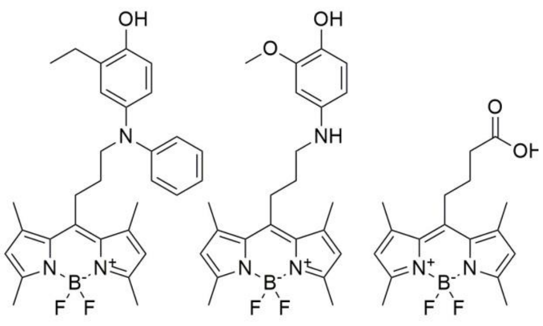

AFG-1 daughter probe control-BODIPY

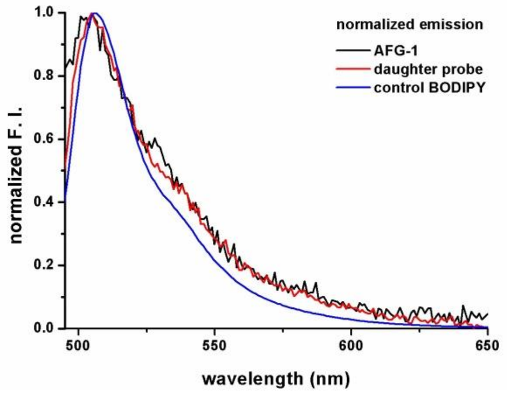

	UV (λmax, nm)	ε (cm^2/mol)	Emission (λmax, nm)	Φ
**AFG-1** (pH 7.4)	515	14 820	505	0.017

3. Lakowicz JR. Principles of Fluorescence Spectroscopy (Third Edition) **2006**, Springer, Newyork

**AFG-1** (pH 4.0)	515	14 500	505	0.025
Daughter probe (pH 7.4)	496	13 360	505	0.074
Daughter probe (pH 4.0)	495	12 630	505	0.133
Control probe	495	66 030	505	0.800

Supplementary Figures

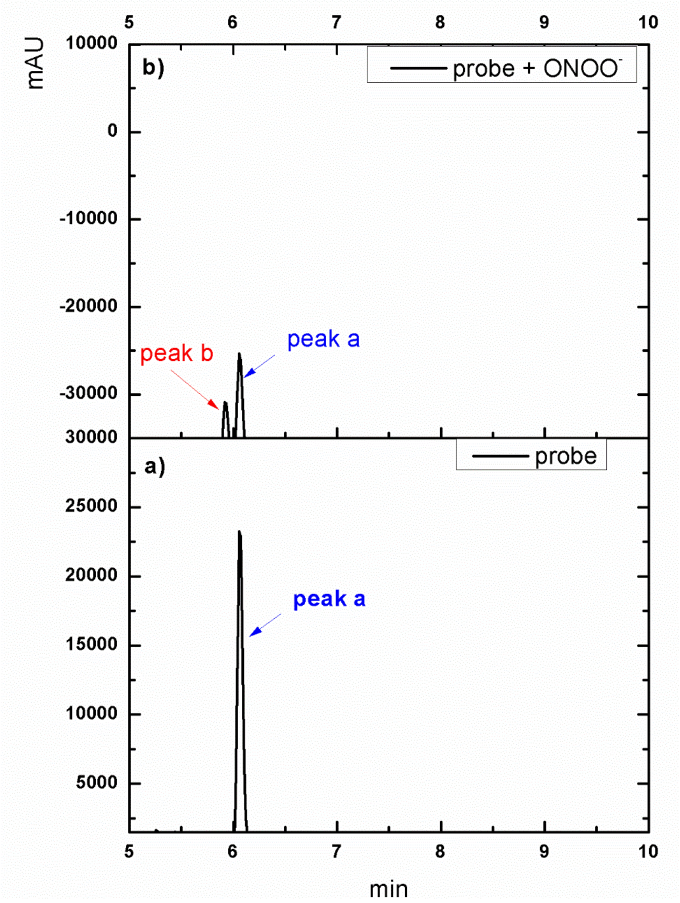

Figure S1. Liquid chromatography (LC) traces of **AFG-1** (20 μM) before (a) and after (b) the treatment of $ONOO^-$ (40 μM). The reaction was carried out in PBS (10% EtOH, 100 mM, pH 7.4) at ambient temperature. The UV-Vis monitor of the LC instrument was set at 254 nm.

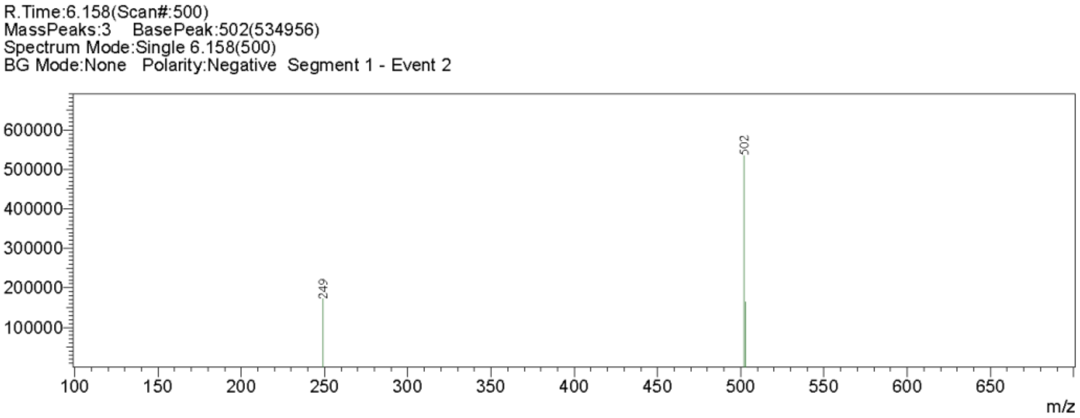

Figure S2. MS spectra trace ([M-H]⁻) of peak a in Figure S1, which is corresponding to structure of **AFG-1**.

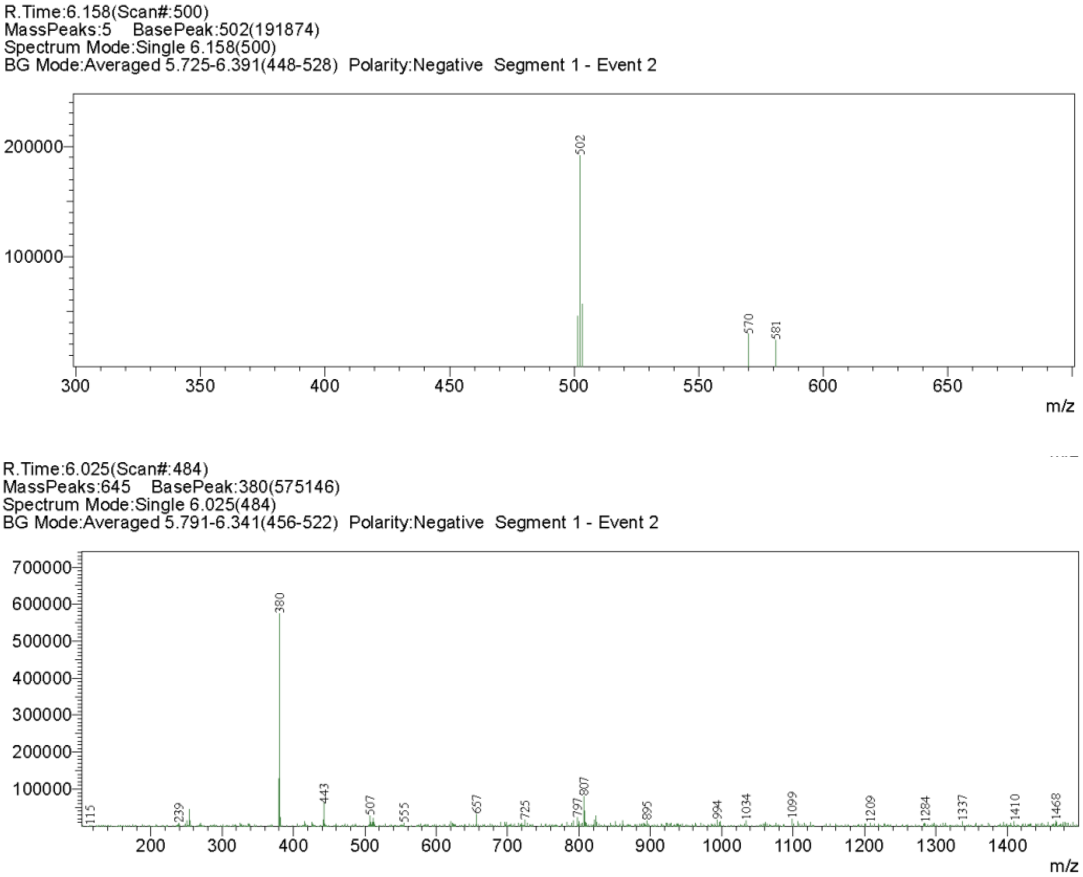

Figure S3. MS spectra trace ([M-H]⁻) of peak a (top) and b (bottom) in Figure S1, respectively corresponding to **AFG-1** and the daughter probe.

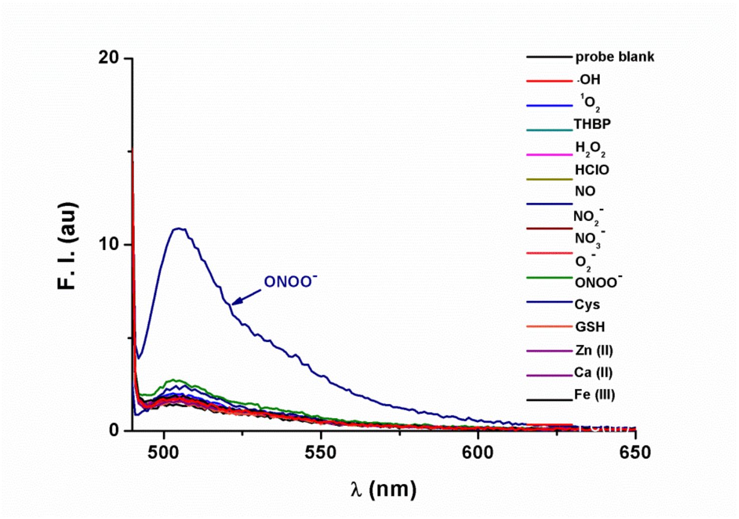

Figure S4. Fluorescence spectra of **AFG-1** (5.0 μM) after being treated with various biologically relevant species (10 μM). Data were obtained in PBS (pH 7.4, 10% EtOH, 100 mM) at ambient temperature (λex 488 nm).

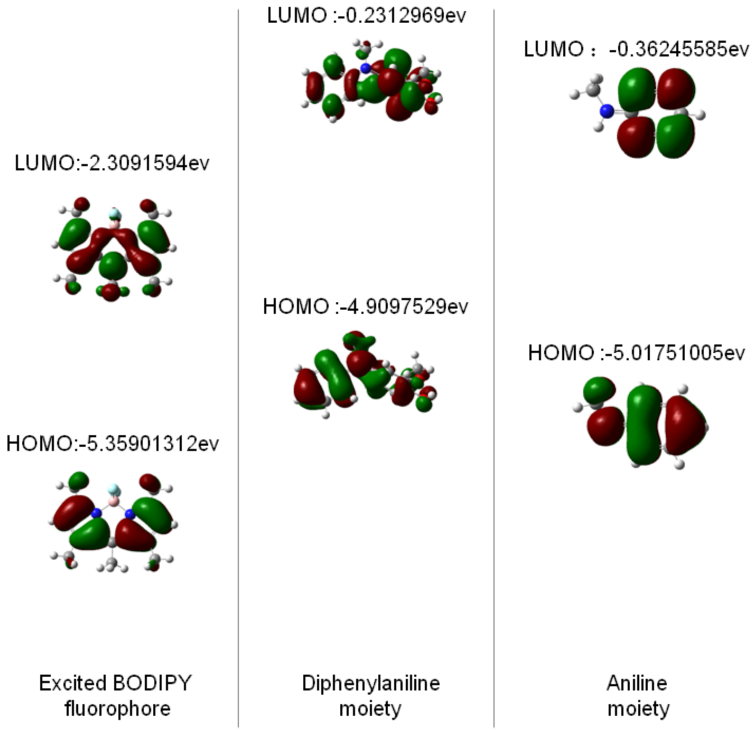

Figure S5. Frontier molecular orbital energies of the excited BODIPY fluorophore, the diphenylaniline moiety, and the aniline moiety calculated by Gaussian using B3LYP method. The results showed an a-PeT fluorescence quenching mechanism.

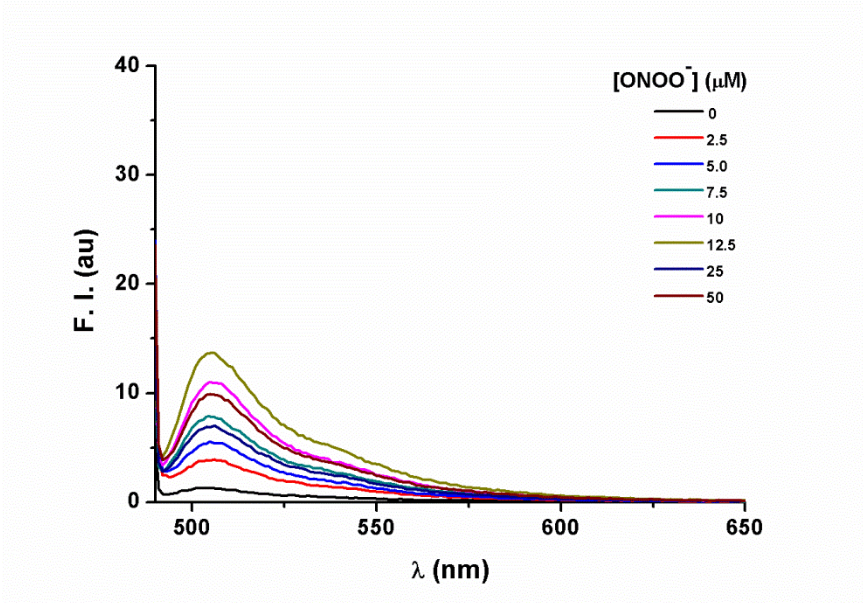

Figure S6. Full spectra overlay of **AFG-1** (5.0 μM) after reacting with $ONOO^-$ of various concentrations at pH 7.4 in PBS (10% EtOH, 100 mM) at ambient temperature (λex 488 nm).

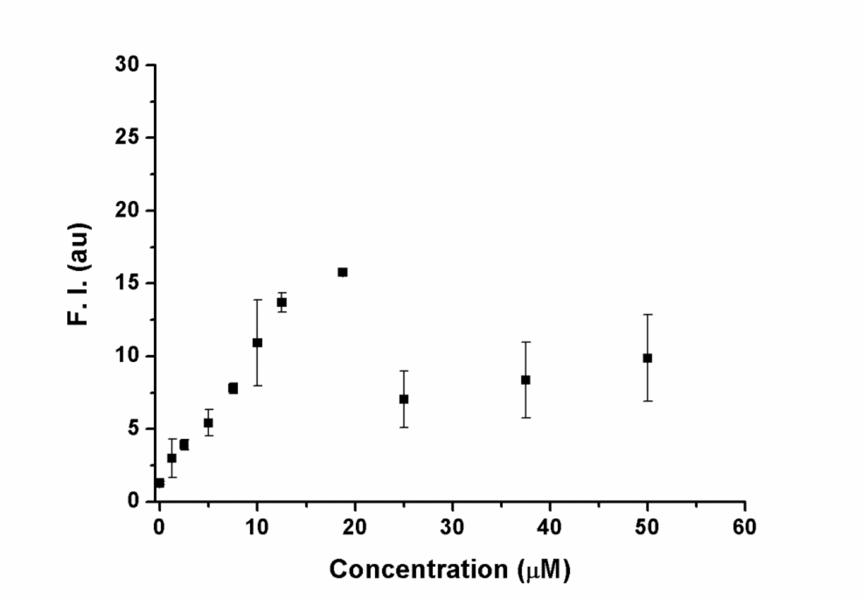

Figure S7. Concentration titration course at 505 nm of AFG-1 (5.0 μM) with $ONOO^-$. Dara shown were collected in PBS (10% EtOH, 100 mM, pH 7.4) after 30 min of incubation.

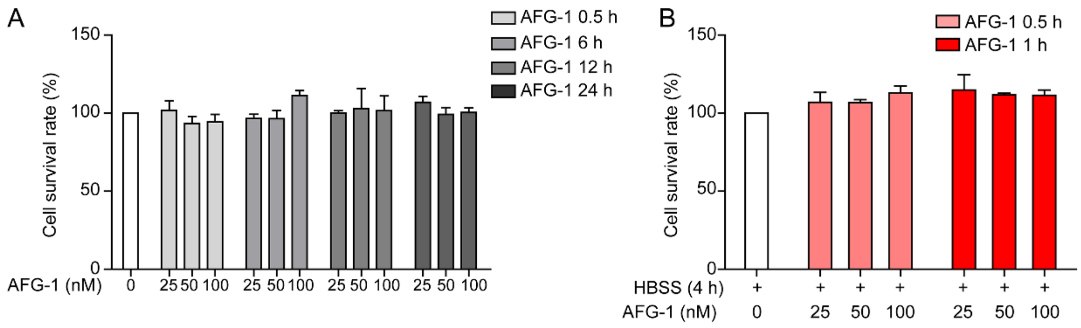

Figure S8. Cytotoxicity evaluation of AFG-1 in EA. hy926 cells without or with HBSS treatment. A) EA. hy926 cells were incubated with various doses of **AFG-1** (0 nM, 25 nM, 50 nM, 100 nM) for various time points (0.5 h, 6 h, 12 h, 24 h). Cell viability was then detected by CCK8 assay. **B)** EA. hy926 cells were first cultured in HBSS for 4 h to initiate autophagy and then were incubated with various doses of **AFG-1** (0 nM, 25 nM, 50 nM, 100 nM) for various time points (0.5 h, 1 h). Cell viability was detected by CCK8 assay.

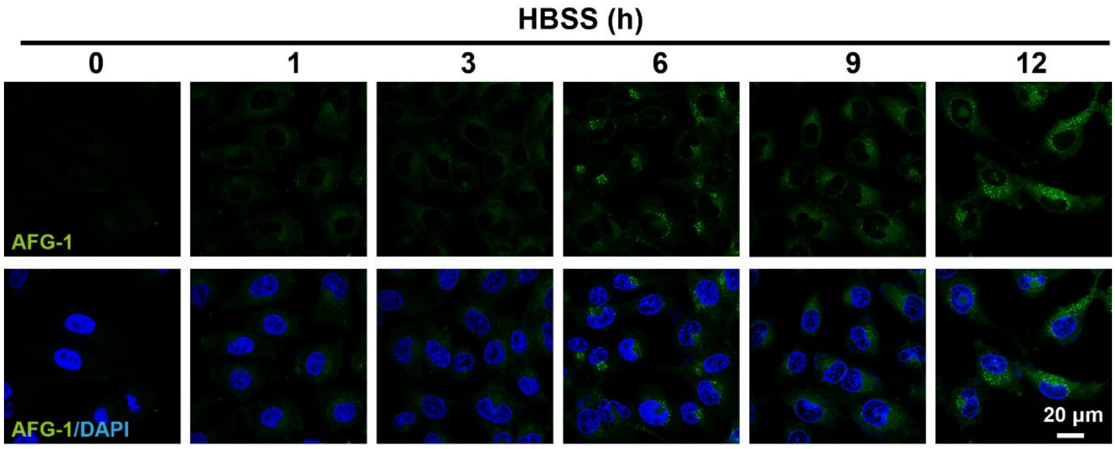

Figure S9. AFG-1 is a sensitive autophagic flux probe in endothelial cells. Representative confocal images show temporal changes of **AFG-1** fluorescence at indicated time points following starvation in EA.hy926 cells. Cells were incubated with 50 nM **AFG-1** probe (green, λ_{ex} 488 nm. λ_{em} 505-550 nm) for 30 minutes and then were imaged by confocal microscopy. DAPI (blue, λ_{ex} 405 nm. λ_{em} 420-480 nm), scale bar: 20 μm.

Supplementary Movies

Movie S1. Real-time visualization of autophagy using probe AFG-1 in ischemic living mice. Real-time visualization of autophagy using *in vivo* two-photon laser scanning microscopy in live mice with brain microvessel injury. A combination of probe **AFG-1** (green, λ_{ex} 488 nm. λ_{em} 505-550 nm) and adenovirus-mRFP-LC3 staining (red, λ_{ex} 543 nm. λ_{em} 560-615 nm) was used for fluorescent imaging. For two-photon imaging, 200 µm below the cortical surface were selected for imaging. The time-series images are individual frames from a continuous time-lapse movie and show dynamic **AFG-1** and adenovirus-mRFP-LC3 fluorescence were elevation compared with the sham mice. Scale bar = 10 µm.

Movie S2. Negative control experiment for movie S1 where mice without brain microvessel injury were imaged.

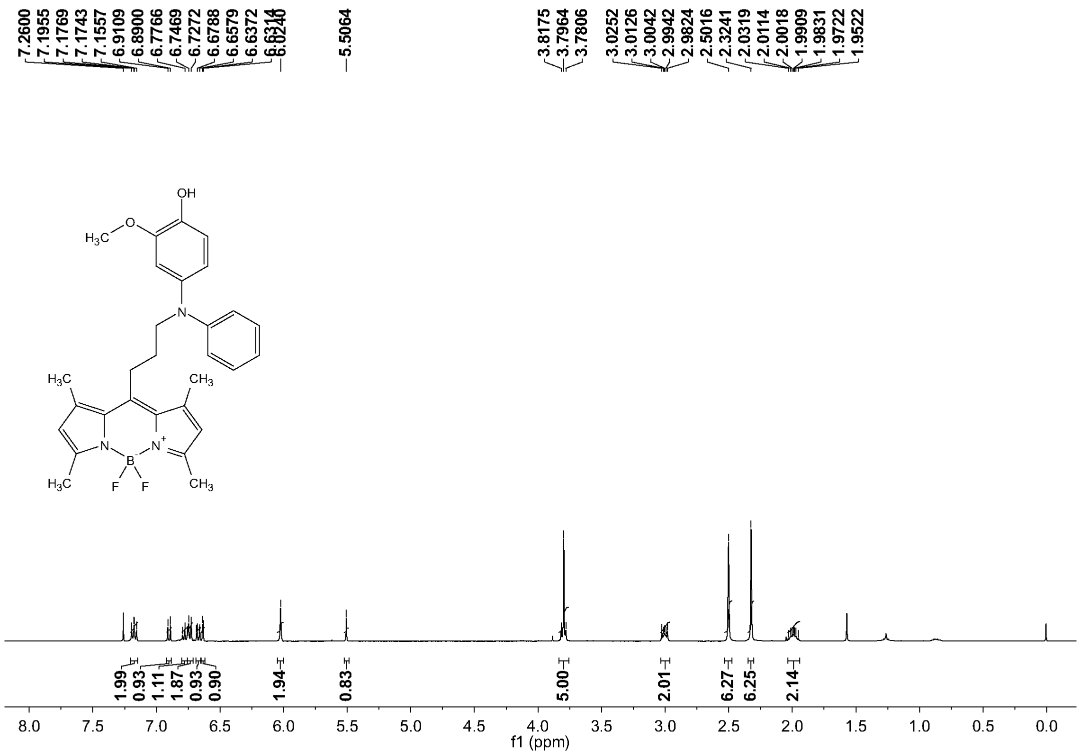

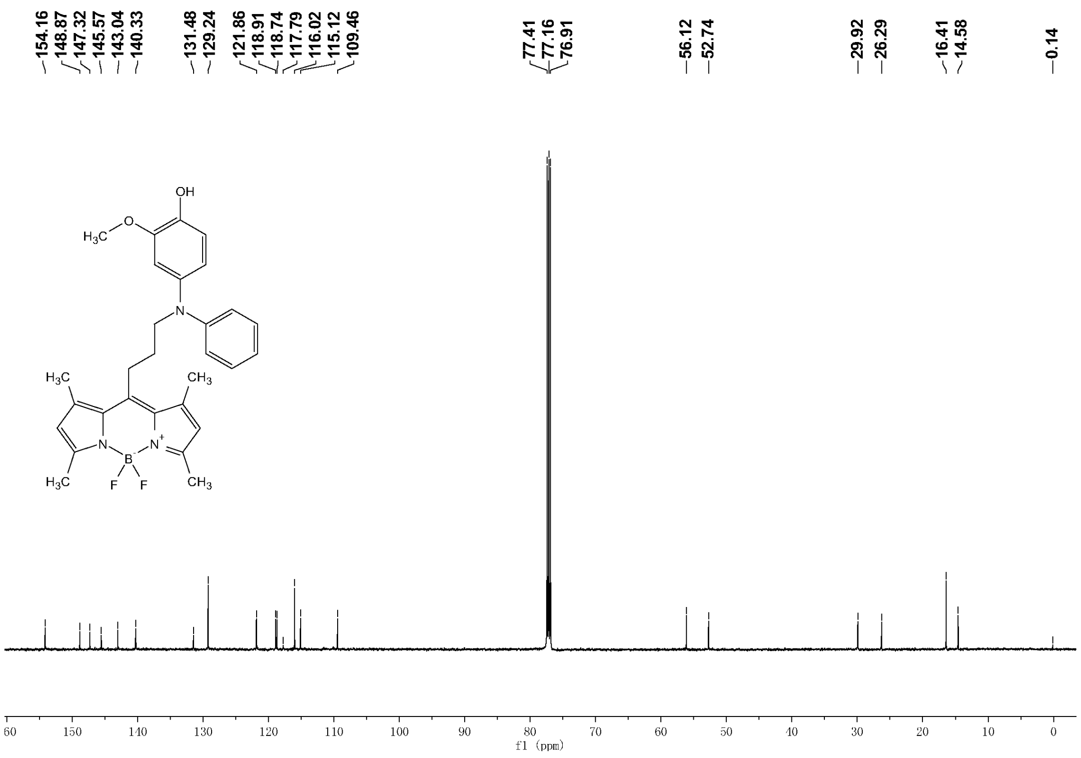

^{1}H NMR and ^{13}C NMR spectra of AFG-1

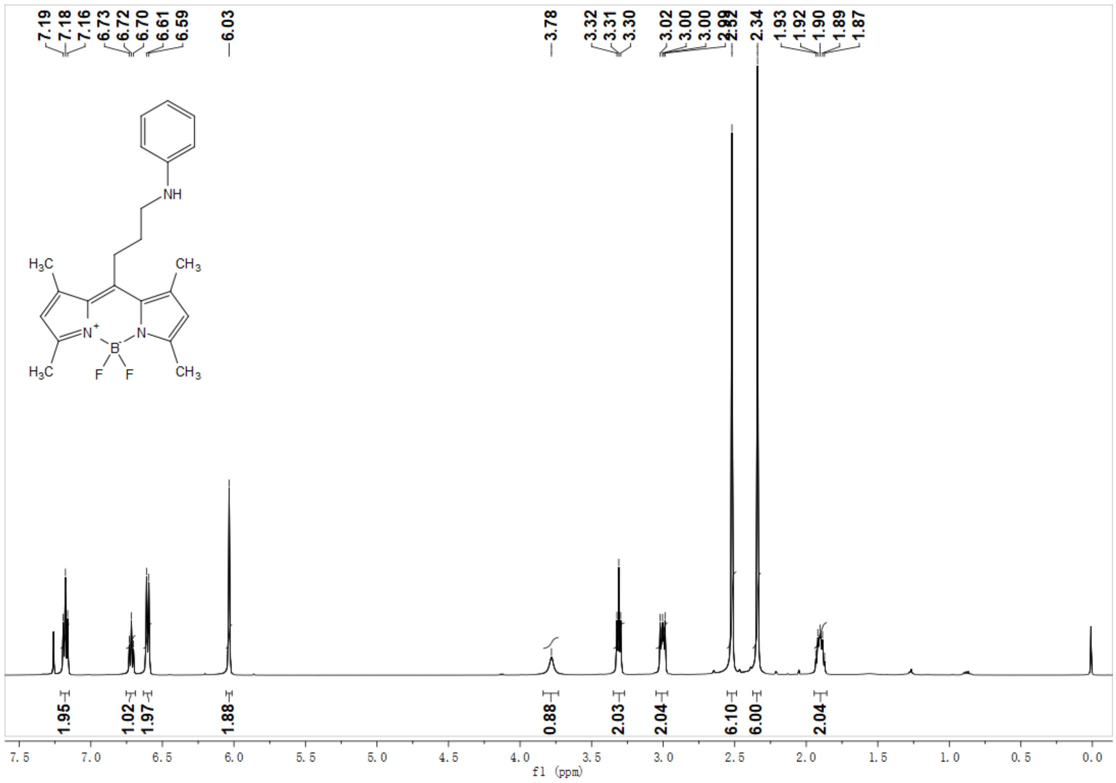

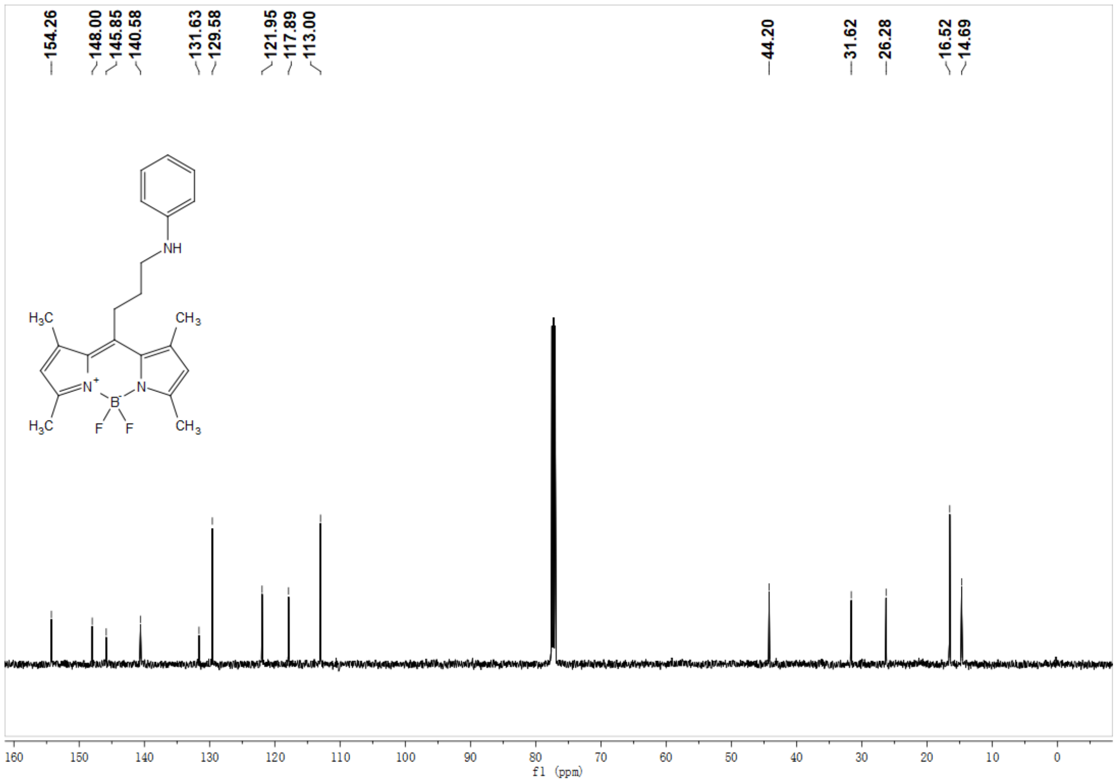

^{1}H NMR and ^{13}C NMR spectra of the daughter probe.
